# Journal indexation and predatory publishing: Publication literacy considerations for family medicine clinicians in Malaysia - A narrative perspective

**DOI:** 10.51866/rv.26-00002

**Published:** 2026-10-23

**Authors:** Jazlan Jamaluddin, Sheron Sir Loon Goh, Hooi Chin Beh, Karleen Chong, Ranita Hisham Shunmugam

**Affiliations:** 1 Department of Primary Care Medicine, Faculty of Medicine, Universiti Malaya, Kuala Lumpur, Malaysia.; 2 Department of Clinical Pharmacy and Pharmacy Practice, Faculty of Pharmacy, Universiti Malaya, Kuala Lumpur, Malaysia.; 3 Department of Library and Information Science, Faculty of Arts and Social, Universiti Malaya, Kuala Lumpur, Malaysia.

**Keywords:** Peer review, Ethics, Publication, Predatory journals, Primary health care

## Abstract

**Introduction:**

Family medicine clinicians rely on published literature for clinical decision-making while facing growing expectations to publish for career progression and postgraduate training requirements. Although journal indexation is often used as a proxy for journal quality, this assumption is misleading given the rise of predatory publishing and evolving digital technologies. This narrative perspective discusses publication literacy considerations for family medicine clinicians in Malaysia, drawing primarily on general scholarly publishing guidance.

**Methods:**

Guidance from indexing organisations and publication ethics bodies was considered alongside selected scholarly literature and Malaysian resources. Discussion was organised around indexation, journal metrics, deceptive publishing practices and practical training. Information sources included Web of Science, Scopus, the National Library of Medicine, the Directory of Open Access Journals, the Committee on Publication Ethics, the International Committee of Medical Journal Editors and materials from Think. Check. Submit.

**Results:**

Web of Science, Scopus and the Malaysian Citation Index have different coverage and purposes; their inclusion decisions do not guarantee article quality. Quartiles describe relative citation performance within a specified category and year. Indexing and metric claims should be checked through official sources alongside editorial policies, fees and journal suitability. General evidence about predatory publishing and artificial intelligence cannot establish the extent of these problems among Malaysian family medicine clinicians.

**Conclusion:**

Publication literacy can be incorporated into family medicine research training through supervised journal comparisons, documented verification before submission and collaboration with medical librarians. These proposed practices require evaluation in Malaysian postgraduate and departmental settings.

## Introduction

Family medicine clinicians substantially rely on published literature to inform clinical decisionmaking, continuing medical education, local guideline development and research. In daily practice, they depend on published evidence to guide diagnostic reasoning, therapeutic decisions, preventive strategies and shared decision-making with patients.^1^ Published literature also underpins continuing professional development, quality improvement initiatives and local or institutional clinical guideline formulation. Simultaneously, many family medicine specialists, particularly those affiliated with universities or teaching hospitals, are expected to contribute scholarly output as part of professional advancement, postgraduate supervision and academic service. Within this dual role as readers and authors, journal legitimacy becomes a matter of practical and professional importance.

The expansion of digital publishing and open-access models has widened opportunities for dissemination, particularly for researchers in low- and middle-income countries.^2^ However, it has also allowed the proliferation of predatory journals to exploit and mimic the appearance of legitimate scholarly publishing while offering limited or non-genuine peer review, editorial oversight or ethical safeguards.^3^ These journals capitalise on academic pressure to publish, rapid turnaround expectations and limited familiarity with publishing standards. Although awareness of predatory publishing has increased over the past decade, practical literacy on how to identify, verify and critically appraise journals among clinicians remains variable.

Journal indexation is frequently used as a proxy for credibility. Inclusion in recognised databases is often interpreted as evidence of quality assurance, ethical standards and editorial rigour. The assumption that an indexed journal is inherently trustworthy is understandable, but it is increasingly problematic.^4^ Indexation systems were never designed to function as absolute quality guarantees. They represent curated inclusion decisions made at a particular timepoint, based on evolving criteria rather than the methodological robustness of individual articles.

This perspective focuses on publication literacy for family medicine clinicians in Malaysia. In this article, resource constraints refer to limited access to research funding, supervision, library support or training within a particular service or institution, rather than a national income category. The aim is to explain the uses and limitations of indexation and journal metrics and propose practical applications for Malaysian family medicine training. Most supporting sources concern scholarly publishing generally; their application to this audience is interpretive rather than evidence of a distinct Malaysian- or family medicine-specific risk.

## Methods

This narrative perspective was informed by selected guidance and policy documents from major indexing organisations and scholarly publishing bodies, together with recent peer-reviewed literature on predatory publishing and vulnerability among early-career researchers. Key sources included Web of Science (WoS) and Clarivate documentation, Scopus selection policy descriptions, National Library of Medicine (NLM) guidance on MEDLINE selection, Directory of Open Access Journals (DOAJ) transparency standards, Committee on Publication Ethics (COPE) guidance on predatory publishing, International Committee of Medical Journal Editors (ICMJE) statements on predatory and pseudo journals and Think. Check. Submit. materials. Malaysian sources included national indexing information and university library guidance.

Sources were selected for their relevance to journal evaluation, the authority of the issuing organisation and their contribution to the discussion of publishing risks. The discussion combines these sources thematically under indexation, quartiles, predatory publishing, artificial intelligence (AI) and educational applications. This perspective is a purposive, non-systematic synthesis and does not claim comprehensive coverage. A reproducible search and screening record, including the number of records identified and excluded, is not available for reporting; therefore, no search yield or exhaustive search period is asserted. Organisational guidance describes publishing standards, whereas empirical studies and commentaries provide different levels of support for claims about author behaviour. The proposed family medicine applications are educational recommendations, rather than findings from a Malaysian clinician cohort.

### Journal indexation

Indexation refers to the inclusion of a journal in a bibliographic or citation database. Selection criteria and coverage differ across systems and may address editorial governance, peer review, publishing ethics, transparency and technical requirements. Indexation status can change, and coverage may be limited to particular collections or publication years.^5^

Major international databases periodically re-evaluate journals and may delist titles that no longer meet expectations. Editorial standards can deteriorate over time; publishers may change ownership; and journals may adopt questionable practices after initial acceptance.^6^ For clinicians, this means that indexation status should be interpreted as provisional rather than definitive.

A common cause of misunderstanding is the distinction between search platforms and curated indexes. PubMed functions as a search interface containing multiple content streams, whereas MEDLINE represents a subset of journals selected through a formal scientific review process. Journals may appear in PubMed without having undergone MEDLINE-level evaluation. Failure to recognise this distinction can cause clinicians to overestimate the methodological rigour of journals encountered during routine literature searches.^7^

Global citation databases such as WoS and Scopus are widely used internationally for benchmarking, research evaluation and academic progression. Their journal selection processes emphasise editorial governance, peer review integrity, citation performance and publication ethics compliance, with ongoing monitoring and the possibility of journal removal if standards are compromised.^8^ Nevertheless, these databases evaluate journals at the structural level and do not guarantee the quality of every published article.

The DOAJ serves a different purpose from major citation databases such as WoS and Scopus. It specifically evaluates open-access journals based on transparency, licensing clarity, archiving practices and ethical publishing policies. Inclusion indicates that minimum standards for openness and governance are met, but it does not replace critical appraisal of individual articles.^9^ The DOAJ does not assign citation-based rankings, while inclusion in WoS or Scopus does not necessarily indicate compliance with open-access best practices; thus, the DOAJ complements but does not replace major citation databases.

At the national level, the Malaysian Citation Index (MyCite), developed under the Ministry of Higher Education through the Malaysian Citation Centre, is a selective bibliographic and citation index for Malaysian scholarly journals. It supports the visibility and evaluation of locally published research and provides national citation data. Its scope therefore differs from that of global citation databases such as WoS and Scopus. Similar to other journal-level indexes, MyCite does not guarantee the methodological quality of each article. Clinicians should verify current MyCite status through the official portal and determine whether it satisfies the requirements of their institution or postgraduate programme.^10^

### Journal quartiles

Journal quartiles are frequently used as shorthand indicators of journal standing, particularly in academic promotion, grant application and postgraduate evaluation. Quartiles are derived from citation-based rankings within subject categories, most commonly through Journal Impact Factor data in WoS or CiteScore metrics in Scopus. Journals are grouped into quartiles, with Q1 representing the top 25% within a category and Q4 the bottom 25%.^11^

Although quartiles are often interpreted as a proxy for quality, this interpretation warrants caution. Quartile position reflects relative citation performance within a defined field rather than an individual article’s methodological rigour or a peer review’s integrity for every published paper. Citation behaviour varies substantially across disciplines. Family medicine, as a broad, clinical practice-oriented and community-focused field, typically generates lower citation rates than laboratory-based or subspecialty disciplines.^12^ Consequently, quartile comparisons across disciplines or even within heterogeneous subject categories may obscure rather than clarify scholarly value.

Quartiles are also vulnerable to misrepresentation. Predatory journals frequently advertise fabricated or misleading quartile claims, sometimes borrowing terminology from legitimate ranking systems without being indexed in the relevant databases.^13,14^ This underscores the necessity of verifying quartile information directly within WoS or Scopus rather than relying on claims displayed on journal websites.

For family medicine clinicians, publication in a lower-quartile journal does not imply poor- quality work, nor does publication in a higher-quartile journal guarantee clinical relevance or methodological robustness. Quartiles are better understood as journal visibility indicators and citation density rather than intrinsic scientific merit.

For early-career clinicians and postgraduate trainees, journal selection should be strategic rather than driven by aspirations to publish in a particular quartile. Publishing in a reputable, appropriately indexed, discipline-aligned journal, even if it is not Q1, is often more academically defensible and professionally sustainable than pursuing an ill-suited high-quartile journal or submitting to a deceptive outlet that promotes false rankings.

### Factors associated with publishing in predatory journals

Publishing in predatory journals is rarely perceived as deliberate misconduct. Rather, it is often the result of structural pressures, knowledge gaps and behavioural vulnerabilities that disproportionately affect clinicians, particularly in resource-limited settings. The sources discussed below span different disciplines and countries and do not establish the frequency or relative importance of these factors among family medicine clinicians in Malaysia.

#### 1. Academic pressure and performance metrics

Early-career clinicians are particularly vulnerable to the pressures of the ‘publish or perish’ culture.^15^ Expectations linked with academic promotion, postgraduate completion, grant eligibility or specialist credentialing can create urgency to publish within short timelines.^16^ When publication quantity is emphasised over quality, clinicians may prioritise speed and acceptance rate over journal legitimacy. Rapid review promises and guaranteed acceptance are hallmark features of predatory outlets and can therefore become attractive under deadline pressure. This is a plausible application to family medicine rather than a demonstrated association among Malaysian trainees.

#### 2. Limited publication literacy

Studies of researchers outside Malaysian family medicine discuss gaps in knowledge about journal selection processes, indexation frameworks, citation metrics and peer review standards. This increases the likelihood that early-career clinicians may inadvertently submit to predatory journals.^17^ Authors who equate a professional online presence with legitimacy or who assume that all open-access journals adhere to comparable editorial standards are particularly vulnerable. Confusion between search platforms and selectively curated indexes further amplifies this risk, leading to overestimation of journal credibility.

#### 3. Early-career status

Early-career clinicians, postgraduate trainees and first-time authors are consistently identified as higher-risk groups. This may be due to limited experience interpreting journal metrics, pressure to establish publication records quickly and a lack of mentorship from senior colleagues.^18^ In some cases, they may overestimate the credibility or prestige suggested by unfamiliar impact indicators or misleading quartile claims promoted by questionable journals. Aggressive email invitations that personalise messages or reference an author’s previous work can further exploit these vulnerabilities and create a false sense of legitimacy.

#### 4. Resource constraints

In low- and middle-income country contexts, access to institutional research support, experienced supervisors or formal research training may be limited.^19^ Financial constraints may also influence decision-making. While predatory journals often charge substantial article processing fees, some offer comparatively lower fees or flexible payment structures, which may appear appealing to authors with limited funding.

#### 5. Inadequate institutional safeguards

Where universities or training programmes do not clearly define acceptable indexation standards, authors may rely on informal advice or peer practices.^17^ In the absence of transparent institutional policies, decisions about journal selection may be shaped more by anecdote, perceived ease of acceptance or prior peer experience than by objective verification of editorial quality. Ambiguity in promotion criteria, particularly when emphasis is placed on numerical output, can inadvertently incentivise submission to questionable journals. Family medicine programmes could reduce this ambiguity by providing written journal selection requirements and identifying whom trainees should consult about uncertain claims.

### Predatory publishing and its evolvement in the AI era

The COPE describes predatory publishing as prioritising profit over quality, with journals charging fees while failing to provide legitimate editorial and publishing services.^20^ The ICMJE has also warned about fake, predatory and pseudo journals and explicitly places responsibility on authors to evaluate the integrity, history, practices and reputation of journals before submission while acknowledging that no single mechanism perfectly identifies all predatory entities.^21^ Peer-reviewed biomedical literature similarly explains that predatory journals attempt to deceive authors by offering publication for a fee without robust peer review or editorial services.^22-24^

Predatory journals are characterised not by geography or access model but by deceptive practices that misrepresent peer review, obscure fees, fabricate metrics or falsely claim indexing.^22,25^ Historically, such journals could often be identified through poor language, crude websites or obvious inconsistencies. This is no longer reliable. Generative AI now enables the rapid creation of polished journal websites, fabricated editorial content and convincing but misleading policy documents. Academic library guidance warns that fake journals may impersonate legitimate publications and misuse journal titles or ISSNs.^26,27^ Recent analyses describe how generative AI has facilitated fraudulent scientific content, including AI-assisted paper mills and deceptive journal operations.^28-32^

Another recurring problem in academic medicine is the conflation of an ‘international’ label with quality. International-sounding titles and glossy websites can be used by journals that are not indexed anywhere meaningful. This issue is particularly relevant when clinicians, including some within family medicine, publish in so-called international but non-indexed journals, potentially assuming that the label alone confers academic weight. By contrast, a reputable, appropriately indexed, discipline-aligned journal may offer greater academic value, more reliable peer review and better discoverability. The practical implication is that clinicians and trainees can no longer rely on website appearance or professional tone as indicators of legitimacy. Verification must instead be anchored in trusted infrastructure, including official index databases and transparent editorial governance (Table 1). The COPE has similarly highlighted evolving patterns of deception associated with technological change.

**Table 1:** Key differences between Web of Science, Scopus and MyCite.

Feature	Web of Science	Scopus	MyCite
Primary scope	International citation database with selective journal evaluation	International abstracting and citation database with curated coverage	National bibliographic and citation database focused on Malaysian journals
Provider	Clarivate	Elsevier	Ministry of Higher Education Malaysia through the Malaysian Citation Centre
Journal selection	Formal evaluation using defined quality criteria; selection is selective.	Selection policy overseen by the Content Selection and Advisory Board	Periodic national evaluation of eligible Malaysian scholarly journals
Ongoing monitoring	Re-evaluation occurs; journals can be de-listed for failing criteria.	Journals are periodically re-evaluated; titles can be discontinued for concerns.	Status is tied to national evaluation cycles and current official listings; need rechecks
Peer review expectations	Mandatory	Mandatory	Expected but variable
Quartile assignment	Yes, by subject category	Yes, by subject category	Not applicable
Use in postgraduate evaluation	Commonly required	Commonly required	Often accepted as a national indexed option, depending on institutional rules
International recognition	High	High	Primarily national, while increasing the visibility of Malaysian scholarship
Risk to early-career authors	Low	Low	Low in national context
Verification of coverage	Clarivate Master Journal List (mjl.clarivate.com)	Scopus Sources (scopus.com/sources)	MyCite (mycite.mohe.gov.my) and Malaysian Citation Centre resources (mysitasi.mohe.gov.my/portal)

Note: These are indexing or citation systems; predatory publishing describes deceptive practices and is therefore discussed separately in Box 2. Coverage does not establish a quantified ‘low risk’ for authors, and non-indexation alone does not establish predatory practice. MyCite: Malaysian Citation Index.

### Implications for family medicine

Early-career clinicians and postgraduate trainees are disproportionately affected by predatory publishing because of limited experience, variable mentorship and strong pressure to publish. A recent *Lancet* commentary highlights this vulnerability and the targeted tactics by predatory publishers to exploit junior academics.^33^ The adverse consequences may become apparent only later, when publications are excluded from thesis requirements or discounted during promotion or academic evaluation. Educational analyses consistently frame this problem as a systems and training gap rather than an individual failing, reinforcing the need for structured curricular responses.

For early-career clinicians, journal selection carries long-term implications. Early publications influence scholarship applications, doctoral opportunities, grant competitiveness and academic appointments. In Malaysia, postgraduate requirements explicitly distinguish between journals indexed in WoS, Scopus or MyCite, meaning that the database in which a journal is indexed may determine whether a manuscript fulfils graduation criteria. In this context, structured verification of indexation is no longer optional but essential. With increasingly sophisticated deception, authors should verify indexation through official sources and apply a checklist approach such as Think. Check. Submit.^34^

Postgraduate programmes and family medicine departments could incorporate a short journal verification session into existing research methods teaching, followed by a supervised comparison of two journals relevant to a trainee’s project. For example, a trainee studying multimorbidity management in Malaysian primary care could compare journal scope, intended readership, recent relevant articles, verified coverage, peer review policies and total publication fees. The trainee would submit a one-page justification of the preferred journal, including the date and official sources checked. This is an illustrative teaching exercise, rather than an evaluated intervention.

Indexation also determines whether research is meaningfully discoverable. PubMed and MEDLINE remain primary access routes for clinicians and researchers to find biomedical literature, and the NLM provides formal mechanisms to verify a journal’s indexing status via the NLM Catalog. Work that is not indexed in major databases may be less readily retrieved and, consequently, less likely to be read, cited or considered in systematic reviews and guideline development. However, these outcomes also depend on the article’s relevance, methodological quality and accessibility, as well as the search strategy used. Therefore, this perspective does not establish a causal relationship between indexation and clinical impact.

### Think. Check. Submit.

Think. Check. Submit. is a global initiative designed to help researchers identify trusted journals using a structured checklist approach. It is intended as a practical tool to evaluate whether a journal is an appropriate venue for a manuscript (Box 1). Its principal strength lies in encouraging authors to apply systematic verification questions rather than depend on informal heuristics. It can be incorporated into research methods teaching but should not be treated as a diagnostic test that conclusively classifies a journal. The ICMJE explicitly emphasises author responsibility in evaluating journal legitimacy and encourages the use of available guidance and mentorship to identify reputable journals.^21^

Box 1 **Think. Check. Submit. adapted for postgraduate trainees and early-career clinicians.**

**Think**

- Is the journal aligned with your work, your discipline and your academic requirements, rather than mainly offering rapid acceptance or frequent unsolicited invitations?

**Check**

- Do you or your colleagues know the journal?
- Is the publisher a current member of a recognised industry initiative?
- Can you verify the journal’s indexing directly through official sources such as Clarivates Master Journal List for WoS coverage or the NLM Catalog for MEDLINE status?
- Does the journal clearly state peer review, editorial oversight, fees, archiving and publishing ethics, consistent with transparency and best practice standards?

**Submit**

- Only submit when the journal’s legitimacy can be justified using verifiable evidence.

### Role of medical librarians in strengthening publication literacy

Medical librarians are critical yet often under-recognised stakeholders in safeguarding publication integrity, particularly in the context of evolving generative AI-driven publishing environment.^35,36^ Building on this role, in the context of generative AI, medical librarians are increasingly positioned to support researchers in interpreting journal policies related to AI-assisted writing, authorship disclosure and ethical use of AI tools, as outlined by major publishing and ethics bodies (ICMJE and COPE).^37-39^ They possess specialised expertise in bibliographic databases, journal indexation systems, citation tracking and the critical evaluation and verification of information sources, particularly in the context ofjournal selection and predatory publishing.^40^ Their role extends beyond literature retrieval to include guidance on journal selection, indexation verification, publication literacy training, research visibility strategies and responsible AI use in scholarly communication. Medical librarians are uniquely positioned to help clinicians and trainees distinguish between search platforms and curated indexes, thereby reducing misinterpretation of a journal’s credibility. This positions librarians not merely as support staff but as institutional gatekeepers of research integrity within clinical academic ecosystems.

Beyond technical verification, medical librarians can contribute meaningfully to structured educational initiatives. These include delivering workshops on journal evaluation and predatory publishing, embedding publication literacy into postgraduate curricula, offering one-to-one consultations during manuscript preparation, supporting citation analysis and advising on institutional publication policies and repository archiving.^41^ In resource-limited settings, where senior mentorship may be limited, librarians serve as accessible and neutral advisors. Integrating librarians into research supervision teams or thesis committees can provide an additional safeguard against inadvertent submission to predatory outlets.

Importantly, involving medical librarians shifts journal verification from an ad hoc individual responsibility to a shared institutional function. Strengthening collaboration between clinicians, academic supervisors and librarians reinforces a culture of research integrity and protects early- career family medicine researchers from avoidable academic setbacks.

### Strengthening family medicine through responsible publishing

Responsible journal selection is not merely an individual concern. It is a collective investment in the credibility of family medicine as an academic discipline. Publishing in indexed, peer-reviewed, discipline-aligned journals increases the likelihood that primary care research is discoverable, citable and incorporated into future evidence syntheses. In family medicine, the most appropriate journals are not always the most famous but the ones that match the discipline’s scope, standards and audience. Historically important discoveries have also first appeared in specialised journals. For example, Alexander Fleming’s original report on penicillin was published in the British Journal of Experimental Pathology in 1929; the discovery of penicillin and its curative effect was later recognised through the 1945 Nobel Prize in Physiology or Medicine.^42,43^ Indexation can facilitate retrieval within a database, but it does not establish the quality of an individual article or assure future coverage. These considerations apply equally to Malaysian and international journals.^44^ In this context, recognising misleading signals and applying structured verification strategies (Box 2) become essential safeguards rather than optional steps. Verification should rely on independent, authoritative sources rather than information presented on journal websites.

Box 2 **Common misleading signals of predatory journals and recommended verification strategies.**

**Misleading signals (red flags)**

- Claims of indexation (e.g. ‘indexed in Scopus/WoS’) without verifiable links
- Fabricated or ambiguous metrics (e.g. ‘Global Impact Factor’ or ‘Universal Impact Score’)
- Misuse of quartile terminology without listing in Journal Citation Reports or Scopus Sources
- Journal titles mimicking established journals or using ‘international’ to imply credibility
- Unrealistically rapid peer review or guaranteed acceptance
- Aggressive or personalised email invitations unrelated to authors’ expertise
- Editorial board members with unverifiable affiliations or listed without consent
- Poorly described peer review, publication ethics or retraction policies
- Inconsistent article processing charges or hidden fees
- Limited publication history warrants further assessment but does not alone establish deceptive practice.

**Verification strategies (recommended practices)**

- Verify indexation directly via Clarivates Master Journal List (WoS), Scopus Sources and/or the NLM Catalog (for MEDLINE/PubMed status).
- Confirm journal metrics only through official databases (JCR or Scopus).
- Verify any claimed COPE membership and, for open-access journals, DOAJ listing; neither guarantees article quality.
- Review past issues for consistency, authorship diversity and article quality.
- Confirm editorial board members through institutional affiliations.
- Apply structured tools such as Think. Check. Submit.
- Seek institutional or librarian support before submission.

### Implications for practice and future directions

This perspective proposes publication literacy as a component of research training for Malaysian family medicine clinicians. Research on predatory journal citations in public health policy documents raises concerns about the trustworthiness of evidence entering policy discussions.^45^ It does not establish the frequency of this problem in Malaysian family medicine or quantify effects on patient outcomes.

For clinical practice, the implications are twofold. First, clinicians must critically interpret published evidence, recognising that indexation and journal metrics are imperfect proxies for quality. Predatory journals often present misleading or false information about editorial processes and metrics, which makes superficial indicators unreliable.^46^ Second, for those involved in research and publication, structured verification of journal legitimacy should be embedded into routine scholarly workflows, as deceptive practices can be difficult to identify without systematic evaluation.

Departments could assess the proposed training through a completed journal verification exercise and a supervisor-reviewed submission plan, rather than attendance alone. A simple assessment could ask trainees to distinguish index coverage from article quality, verify an unfamiliar journal’s identity and explain why its scope fits a primary care study. Programme teams could monitor the completeness of verification records and the accuracy of trainees’ checks before and after teaching. Whether these activities reduce submissions to deceptive journals should be evaluated rather than assumed.^34,47,48^ Integrating medical librarians and structured training into research pathways may help mitigate knowledge gaps and reduce vulnerability among early-career clinicians.

Further Malaysian family medicine research could examine baseline publication literacy, access to supervisory and library support and the feasibility of embedding verification into existing specialist training. There is currently limited empirical consensus on how best to identify predatory journals or measure the effectiveness of detection strategies, highlighting an important gap for further study.^46^

Additional work is needed to understand how predatory publishing continues to evolve, particularly with increasing technological sophistication and the broader pressures of academic publishing systems that prioritise output metrics. Policymakers and academic institutions should also consider clearer and standardised criteria for acceptable indexation to reduce ambiguity in promotion and training requirements. Ultimately, strengthening publication literacy will improve not only academic outcomes but also the quality and trustworthiness of evidence that informs patient care.

### Strengths and limitations

This narrative perspective synthesises organisational guidance and selected scholarly literature to explain journal evaluation for a defined audience: family medicine clinicians in Malaysia. Its practical contribution is a proposed approach to teaching and documenting journal verification within existing research supervision and postgraduate training.

However, several limitations should be acknowledged. First, the source selection was non-systematic and is subject to selection bias; this article is not an exhaustive evidence review. Much of the literature concerns general scholarly communication, other disciplines or other countries. It cannot establish the prevalence, determinants or consequences of predatory publishing among Malaysian family medicine clinicians, so the findings should not be generalised to all resource-constrained settings. Second, predatory publishing is a rapidly evolving phenomenon, with increasingly sophisticated deceptive practices that may outpace existing guidance and frameworks. Third, there is currently no universally accepted definition or standardised method for identifying predatory journals, and existing tools and checklists vary in quality and evidence base. This lack of consensus limits the ability to provide definitive criteria for journal evaluation. Finally, while this review proposes practical strategies for journal verification, the effectiveness of these approaches in real-world clinical and academic settings remains insufficiently studied. Evidence suggests that predatory journals can be difficult to distinguish from legitimate ones, reinforcing the need for further empirical research.^46^

## Conclusion

For family medicine clinicians in Malaysia, journal indexation and quartiles are useful contextual information but not guarantees of article quality or journal suitability. This narrative perspective applies general publication guidance to clinical scholarship and proposes supervised journal comparisons, dated verification before submission and collaboration between trainees, supervisors, programme staff and medical librarians. These practices can be incorporated into existing research teaching, but their feasibility and effectiveness in Malaysian family medicine require evaluation.

## References

[1] Ng CJ, Lee PY (2021). Practising shared decision making in primary care.. Malays Fam Physician..

[2] Newton CR (2020). Research and open access from low- and middle-income countries.. Dev Med Child Neurol..

[3] Elmore SA, Weston EH (2020). Predatory journals: what they are and how to avoid them.. Toxicol Pathol..

[4] Bohannon J (2013). Who’s afraid of peer review?. Science..

[5] Clarivate Web of Science journal evaluation process. Clarivate.

[6] Macdonald S (2025). The scandal of academic publishing.. PublRes Q..

[7] National Library of Medicine MEDLINE selection. https://www.nlm.nih.gov/medline/medline_journal_selection.html.

[8] Elsevier Scopus content policy. https://www.elsevier.com/products/scopus/content.

[9] Directory of Open Access Journals Guide to applying. https://doaj.org/apply/guide/.

[10] Department of Higher Education Keputusan Penilaian Jurnal Ilmiah Malaysia (MyCite) 2025. Ministry of Higher Education Malaysia.

[11] Miranda R, Garcia-Carpintero E (2019). Comparison of the share of documents and citations from different quartile journals in 25 research areas.. Scientometrics..

[12] Declaration on Research Assessment (DORA) San Francisco Declaration on Research Assessment. https://sfdora.org/read/.

[13] Perlin MS, Imasato T, Borenstein D (2018). Is predatory publishing a real threat? Evidence from a large database study.. Scientometrics..

[14] Laine C, Winker MA (2017). Identifying predatory or pseudo-journals.. Biochem Med (Zagreb)..

[15] Isaacs D (2022). Publish, flourish or perish: the ups and downs of journal publication.. J Paediatr Child Health..

[16] Bayanbayeva A (2026). The impact of the ‘publish or perish’ culture on research practices and academic life in Kazakhstan: challenges and consequences in the age of global university rankings.. High Educ Res Dev..

[17] Moshi SS, Dulle F, Tarimo RB (2025). Investigating researchers’ attitude toward predatory publishing in Tanzanian universities.. Inf Dev..

[18] Johann D, Neufeld J, Thomas K, Rathmann J, Rauhut H (2024). The impact of researchers’ perceived pressure on their publication strategies.. Res Eval..

[19] Dakhil ZA, Cader FA, Banerjee A (2024). Challenges in clinical research in low and middle income countries: early career cardiologists’ perspective.. Glob Heart..

[20] COPE Council (2019). COPE Discussion Document: Predatory Publishing. https://publicationethics.org/guidance/discussion-document/predatory-publishing.

[21] International Committee of Medical Journal Editors Recommendations. https://www.icmje.org/recommendations/.

[22] Cobey KD, Lalu MM, Skidmore B, Ahmadzai N, Grudniewicz A, Moher D (2018). What is a predatory journal? A scoping review.. Fl000Res..

[23] Shamseer L, Moher D, Maduekwe O (2017). Potential predatory and legitimate biomedical journals: can you tell the difference? A crosssectional comparison.. BMC Med..

[24] Moussa S (2021). Contamination by citations: references to predatory journals in the peer-reviewed marketing literature.. S Asian J Mark..

[25] Shen C, Bjork BC (2015). ‘Predatory’ open access: a longitudinal study of article volumes and market characteristics.. BMC Med..

[26] Universiti Malaya Library Predatory journals and publishers. Universiti Malaya.

[27] Research Services Division, Perpustakaan Sultan Abdul Samad Predatory journal. Universiti Putra Malaysia.

[28] Lopez-Borrull A, Lopezosa C (2025). Mapping the impact of generative AI on disinformation: insights from a scoping review.. Publications..

[29] Spinellis D (2025). False authorship: an explorative case study around an AI-generated article published under my name.. Res Integr Peer Rev..

[30] Gu J, Wang X, Li C (2022). AI-enabled image fraud in scientific publications.. Patterns (NY)..

[31] Jamaluddin J, Gaffar NA, Din NSS (2023). Hallucination: a key challenge to artificial intelligence generated writing.. Malays Fam Physician..

[32] Lee PY, Salim H, Abdullah A, Teo CH (2023). Use of ChatGPT in medical research and scientific writing.. Malays Fam Physician..

[33] Laine C, Babski D, Bachelet VC (2025). Predatory journals: what can we do to protect their prey?. Lancet..

[34] Think. Check. Submit. https://thinkchecksubmit.org/.

[35] Wattanapisit A, Photia A, Wattanapisit S (2023). Should ChatGPT be considered a medical writer?. Malays Fam Physician..

[36] Wattanapisit A, Wattanapisit S, Mallen C (2026). Affirming generative artificial intelligence as a co-pilot, not a co-author, in medical and scientific writing.. Malays Fam Physician..

[37] Rodzvilla J (2025). Predatory publishing and global scholarly communications.. J Web Librarianship..

[38] International Committee of Medical Journal Editors Use of AI by authors. https://www.icmje.org/recommendations/browse/artificial-intelligence/ai-use-by-authors.html.

[39] COPE Council (2024). COPE position: authorship and AI.

[40] Berger M (2024). Predatory Publishing and Global Scholarly Communications.

[41] Pinto M, Garcia-Marco J, Caballero D, Manso R, Uribe A, Gomez C (2024). Assessing information, media and data literacy in academic libraries: approaches and challenges in the research literature on the topic.. J Acad Librariamhip..

[42] Fleming A (1929). On the antibacterial action of cultures of a penicillium, with special reference to their use in the isolation of B. influenza.. Br J Exp Pathol..

[43] Tan SY, Tatsumura Y (2015). Alexander Fleming (1881-1955): discoverer of penicillin.. Singapore MedJ..

[44] Hani S (2025). Looking back at 2025: strengthening primary care evidence for Malaysia, regional partners, and the global health community.. Malays Fam Physician..

[45] Albert MA, Lalu MM, Grudniewicz A (2025). Investigating the trustworthiness of research evidence used to inform public health policy: a qualitative interview study on the use of predatory journal citations in policy documents.. Health Res Policy Syst..

[46] Pollock D, Barker TH, Stone JC (2024). Predatory journals and their practices present a conundrum for systematic reviewers and evidence synthesisers of health research: a qualitative descriptive study.. Res Synth Methods..

[47] Cortegiani A, Shafer SL (2018). “Think. Check. Submit.” to avoid predatory publishing.. Crit Care..

[48] Frederick DE (2020). Scholarly communications, predatory publishers and publish or perish culture in the 2020s.. Libr Hi Tech News..

